# Effect of Isomixing on Grape Must Fermentations of *ATF1*–Overexpressing Wine Yeast Strains

**DOI:** 10.3390/foods9060717

**Published:** 2020-06-02

**Authors:** Niël van Wyk, Florian Michling, Dennis Bergamo, Sylvia Brezina, Isak S. Pretorius, Christian von Wallbrunn, Jürgen Wendland

**Affiliations:** 1 ARC Centre of Excellence in Synthetic Biology, Department of Molecular Sciences, Macquarie University, Sydney 2113, NSW, Australia; sakkie.pretorius@mq.edu.au; 2 Department of Microbiology and Biochemistry, Hochschule Geisenheim University, 65366 Geisenheim, Germany; florian.michling@hs-gm.de (F.M.); dennis.bergamo@hs-gm.de (D.B.); Silvia.Brezina@hs-gm.de (S.B.); Christian.Wallbrunn@hs-gm.de (C.v.W.); Juergen.Wendland@hs-gm.de (J.W.)

**Keywords:** alcohol acetyl transferase, isomixing, microvinification, *Saccharomyces cerevisiae*, wine yeast, recombination

## Abstract

Speeding up grape must fermentation would be of great economic benefit. We subjected Saccharomyces cerevisiae VIN13 and two recombinant VIN13-strains expressing *ATF1* alleles under two different promoters (either *PGK1* or *HXT7*) to four styles of grape must fermentations; we then assessed the effect of constantly stirring a must fermentation (isomixing). The four different fermentation setups were as follows: *isomixed, closed* in an ANKOM Rf Gas productions system; *isomixed, open* in a stirred tall tube cylinder; *static, closed* constituting a conventional fermentation in a wine bottle equipped with an airlock and static; and *static, open* in a tall tube cylinder (without stirring). We report on major fermentation parameters and the volatile aroma compositions generated in the finished wines. The primary fermentations of the strains subjected to constant stirring finished after 7 days, whereas the static fermentations reached dryness after 19 days. The wines derived from isomixed fermentations produced approximately 0.7% less ethanol compared to the unstirred fermentations. The speed that the isomixed fermentation took to reach completion may provide an alternative to static fermentations in the preparation of base wines for sparkling wine production. The observed increase of volatiles of isomixed fermentations merits further investigation.

## 1. Introduction

In wine research, microvinifications are important tools employed by researchers to investigate all aspect of enology. The overwhelming majority of microvinifications are simply performed either in Erlenmeyer flasks or in wine bottles fitted with airlocks. This occurs with respect to higher throughput in fermentations. Only after promising conditions or candidate strains have been identified are larger volumes fermented to also allow a full sensory analysis via taste panels. A crucial aspect of wine fermentations is to determine when the so-called primary fermentation is finished and has reached dryness, i.e., when the fermentable sugars have been completely consumed. Normally, this requires roughly 1–3 weeks of fermentation, but if problems arise, prolonged fermentations can take more than twice that, or never reach dryness [1]. The high initial sugar concentration of grape must (~200 g/L, about 80° Oechsle), low nitrogen content, suboptimal temperature, low pH, i.e., of ~3.0, the accumulation of ethanol, along with the anaerobic and static nature of the wine fermentation are the main factors that contribute to the time it takes for completion. Providing methods to speed up fermentations or address problematic fermentations without compromising on the quality of the product would be of great economic benefit.

As yeast (mostly *Saccharomyces cerevisiae*) is the main sugar consumer and ethanol producer during wine fermentation, its adaptation and behavior in must is pivotal. Yeasts are also important contributors in imparting flavor to the final wine product, particularly with regards to their higher alcohol and ester production [2,3,4]. Many genes key to aroma compound biosynthesis have been identified [5]; of note are the alcohol acetyl transferase (*ATF*)-encoding genes catalyzing the condensation reaction between an alcohol and acetyl-CoA, forming the acetate ester of the respective alcohol. In S. cerevisiae, there are two paralogous acetyl transferase genes called *ATF1* and *ATF2*. It has been shown repeatedly that by overexpressing the *ATF*-encoding genes (using strong constitutive promoters, such as the promoters of *PGK1*, *TDH3* or *GPD1*), a dramatic increase in the acetate ester content of beverages can be achieved [6,7,8]. This feature has already been introduced in commercial applications, e.g., in Japan, ‘self-cloning’ procedures in yeast strain constructions that yield strains that do not contain foreign DNA are used in the production of saké [9]. The use of genetically modified organisms (GMOs) in winemaking is highly restrictive, with only two wine yeast strains currently being allowed to be used in commercial winemaking [10]. Despite this, wine researchers have been exploring the use of GM yeast in microvinifications for more than 25 years with the aim of building a “treasure trove” of wine yeast with beneficial oenological attributes in the hope of achieving more acceptance in the future [11,12,13].

In this study, we investigated if and how the wine parameters and volatile content are affected by four different fermentation styles using a Müller-Thurgau grape must, simultaneously assessing the utility of *ATF1* expression controlled by either a glucose-sensitive promoter (*HXT7*) or the constitutive *PGK1* promoter.

## 2. Materials and Methods

### 2.1. Plasmid Construction

**Figure 1:**
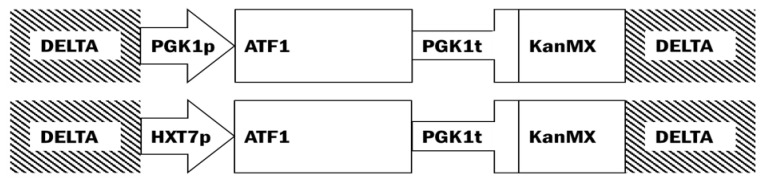
Relevant features of the pBKD1-derived plasmids used in the study. Plasmids were linearized with XhoI endonuclease (located in delta (Ty) genetic elements).

*Escherichia coli* DH5α (Stratagene, La Jolla, CA, USA) was used for plasmid propagation. The *E. coli-S. cerevisiae* shuttle vector, plasmid pBKD1 plasmid [14], was used for genetic manipulation (Figure 1). Flanking the promoter and terminator sequences of *PGK1* of *S. cerevisiae* and the *KanMX* are delta sequences (Ty retrotransposon) of *S. cerevisiae*. The delta sequences can be integrated into the genome of *S. cerevisiae*. The 1578-bp *ATF1* open reading frame (ORF; YOR377w and *HXT7* promoter (1000 bp upstream of ORF YDR342c)) was amplified using Phusion Taq polymerase (Thermo Scientific, Waltham, MA, USA) from genomic DNA of *S. cerevisiae* VIN13 using the primers listed in Table S1. The amplicon containing *ATF1* was cut with two restriction endonucleases, PacI and AscI (Thermo Scientific), and ligated using T4 ligase (Thermo Scientific) into a PacI and AscI digested pBKD plasmid between the promoter and terminator sequences of *PGK1*. The amplicon containing the *HXT7* promoter was digested with PacI and ligated in a PacI-digested pBKD plasmid already containing the *ATF1* gene. Plasmids were confirmed using polymerase chain reaction (PCR) amplification and Sanger sequencing.

### 2.2. Yeast Transformation

The yeast transformation protocol for the wine yeast VIN13 (Anchor Yeast, Cape Town, South Africa) is an adaptation of the protocol described previously using the XhoI-digested pBKD plasmids [15]. Cells were plated on YPD agar containing 0.2 mg/mL G418. The genomic DNA of colonies that appeared after 2–3 days was isolated, and the integration of the cassette into the yeast genome was confirmed with PCR amplification using ScATF1_R with either HXT7prom_F or PGK1prom.

### 2.3. Grape Must

Pasteurised Müller-Thurgau grape must (harvested in 2018 from a vineyard of the Geisenheim University in the Rheingau wine region of Germany) was used in this study. The must had a pH of 3.22 and glucose and fructose concentrations of 93.4 g/L and 100.1 g/L, respectively (total sugars 193.5 g/L). Tartaric acid and malic acid were 5.3 g/L and 1.7 g/L, respectively. Its primary amino acid content was assayed using the NOPA (nitrogen by *o*-phthaldialdehyde) method and was shown to be 70 mg/L. The free ammonium level was 43 mg/L, as determined using a Rapid Ammonium kit from Megazyme (Bray, Ireland). Opti-MUM White^TM^ (Lallemand, Montreal, QC, Canada) was added at a concentration of 20 g/hL to supplement any nutrient deficiencies.

### 2.4. Fermentations

Four fermentation modes were employed to ferment the pasteurized Müller-Thurgau grape must. Precultures of the three VIN13-based strains were prepared by overnight growth in shake flasks at 22 °C using an autoclaved version of the aforementioned must. The must was inoculated at a concentration of approximately 1 × 10^7^ cells/mL (as determined with a hemocytometer) before separating it into the different vessels. The ‘conventional’ static method entailed pouring 220 mL of the inoculated must in a 250 mL wine bottle topped with an airlock, and is designated here as *static*, *closed* fermentation. For both types of the open fermentations, 250 mL tall-tube cylinders were used. Two-hundred-and-twenty milliliters of inoculated must was added into the cylinders. For the *isomixed, open* fermentation, cylinders—each equipped with a magnetic stirrer bar—were placed onto magnetic stirrer pads and continuously stirred at 300 rpm. For the *static*, *open* fermentation, cylinders were used without stirring. All the cylinder tops were loosely capped with aluminum foil to allow air exchange to occur. For the *isomixed, closed* fermentation, 220 mL inoculated must was placed in a modified 250 mL borosilicate bottle capped with a lid which contained the Rf sensor module of the ANKOM Rf Gas Production system (ANKOM Gesellschaft für Analysentechnik—HLS, Salzwedel, Germany). Bottles equipped with an appropriate magnetic stirrer bar were—like with the *isomixed, open* setup—placed on a magnetic stirrer pad, with the must subjected to constant stirring. The setup for the ANKOM was conducted according to the manufacturer’s instructions.

All fermentations took place in a 22 °C incubator and were done in quadruplicate. Wine bottles and cylinders were weighed daily, and the fermentations were stopped once no further weight-loss could be recorded over one day. For the ANKOM system, once the accumulated pressure measured by the Rf sensor reached a plateau, samples were considered finished. Samples were then placed at 4 °C prior to HPLC and GC-MS analyses.

### 2.5. HPLC-Analysis

To determine the final concentrations of the major organic acids, glycerol, sugars and ethanol, high performance liquid chromatography (HPLC) was employed as described previously [16]. An Agilent Series 1100 HLPC, equipped with a binary pump, an autosampler, a multi-wavelength detector (MWD) and a refractive index (RI) detector (Agilent Technologies, Steinheim, Germany) was used.

### 2.6. Volatile Compound Analysis

Wine samples of 5 mL each were subjected to head space-solid phase microextraction gas chromatography mass spectrometry (HS-SPME-GC-MS) analysis to assess the quantities of various aroma compounds. A GC 6890N was equipped with an MS 5977B (both Agilent, Santa Clara, CA, USA) and an MPS II robotic autosampler and CIS 4 (both Gerstel, Mülheim an der Ruhr, Germany). Ten microliters of an internal standard (1-octanol was to 5 mL of a wine sample and 1.7 g of NaCl were placed into a 20 mL head space vial. Solid phase microextraction was carried out with a 65 µm polydimethylsiloxane and divinylbenzol fiber (Supelco, Merck, Darmstadt, Germany). Aroma compound separation was performed using a 60 m × 0.25 mm × 1 µm gas chromatography column (Rxi^®^-5Si1, Restek, Bad Homburg v. d. Höhe, Germany) with helium used as carrier gas. The sample was injected in split mode (1:10, initial temperature 30 °C, rate 12 °C/s to 240 °C, hold for 4 min). The GC run started with an initial temperature of 40 °C for 5 min, which was then raised to 210° C at 5 °C/min, and raised again to 240 °C at 20°/min and held for 10.5 min. Mass spectral data were acquired in a range of mass to charge ratio (m/z) of 35 to 250 and used to derive concentration values. A 5-point calibrations curve was used for each volatile compound within a wine model solution of 10% ethanol with 3% tartaric acid pH 3, as described previously [17].

### 2.7. Data Analysis

Principal Component Analysis (PCA), PCA 1, was conducted on a data table of 26 analytes (% ethanol, amount of glycerol, 4 organic acids and 20 volatiles obtained for four different styles of grape must fermentation with VIN13[REF] run in quadruplicate (*n* = 4 × 4 = 16). Distance biplots showing analyte contribution to the formation of reduced space and sample ordination therein are provided. As for PCA 1, the number of observations (*n* = 16) was smaller than the number of analytes (*n* = 26); estimates of correlation among the latter were nonindependent. To examine potential bias, we performed Iterative Partial Least Squares (NIPALS) on 100 resampled data sets (with replacement) and found that the variable loadings (i.e., analyte contribution to a principal-component axis) obtained in this fashion showed strong correlation to the variable loadings of PCA 1. Thus, we report the results obtained via the ‘classical PCA’ framework. NIPALS was performed as implemented in function nipals() from package ade4 [18].

A second PCA, PCA 2, was performed on the same set of analytes including additional observations from fermentations with genetically modified strains VIN13[HXT7p_ATF1] and VIN13[PGK1p_ATF1] under otherwise equivalent conditions (*n* = 16 + 2 × 4 × 4 = 48).

To account for heterogeneity of measurement unit and differences in order of magnitude among analytes (e.g., % ethanol; µg/L acetate esters and g/L organic acids), all PCAs were performed on scaled data (Z-scores; i.e., PCA of a correlation matrix). Consequently, all analytes contributed equally to total variation.

A redundancy analysis (RDA) was used to perform multivariate analyses of variance (MANOVA) with independent variables being stirring regimen (isomixed or static fermentation) and capping type (closed or open fermentation). Details of the procedure are given in [19,20]. In short, RDA performs multivariate linear regression—in our case, regressing 26 analytes on stirring regimen and capping type—and then PCA on the fitted values, which we do not show here, as the ordination on RDA axes was little different to that obtained by PCA (i.e., fermentation styles were readily separated in PCA space, see below). We used RDA exclusively to obtain estimates of R^2^ for each independent variable, i.e., the proportion of variation which could be attributed to the independent variables.

PCA and RDA were conducted using function rda() from package vegan 2.5–6 [21]. Prior to estimating R^2^ via RDA, multivariate homogeneity of variance among groups (fermentation styles) was tested using functions betadisper() and permutest() from the same package. Significance was assessed by 99 permutations. All analyses were performed in R version 3.6.2 [22], following guidelines detailed in [19,20]. Additional R packages used in generating plots, were: shape [23].

For multiple comparisons, we applied linear contrasts and implemented in function lincon() from R package WRS [24]. For details on the method, refer to Sections 7.4.1 and 7.4.2 in [25]. Linear contrasts constitute a robust way to compare multiple means, and do not require, for example, homogeneity of variance among groups; confidence intervals and *p*-values accommodate a family-wise error rate of 0.05 for each analyte. *p*-values were adjusted using Holm-correction [26].

## 3. Results

### 3.1. Strain Generation

Genetic modifications were performed on a widely-used commercial *Saccharomyces cerevisiae* wine yeast strain, VIN13 in this study. The native *ATF1* gene was placed under the transcriptional control of the strong constitutive promoter of the *PGK1* gene (hereafter denoted VIN13[PGK1p_ATF1]). The promoter of the *HXT7* gene was also used to investigate its transcriptional power under winemaking conditions, with the resulting strain containing the *HXT7* promoter driving *ATF1* transcription denoted as VIN13[HXT7p_ATF1]. Stable integration of these genetic constructs using the retrotransposon Ty (delta) elements was achieved, and of note was the strong banana/pineapple smell detected from the agar plates containing the transformants (especially of VIN13[PGK1p_ATF1]). The generated strains still harbored a *KanMX* resistance marker, and thus, no tastings were conducted on the resulting wines.

### 3.2. Fermentation Performance

#### 3.2.1. Fermentation Speed

**Figure 2:**
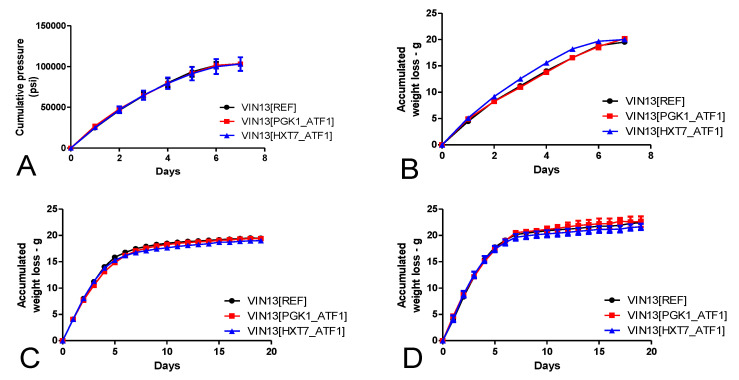
Accumulated weight loss or cumulative pressure of fermentations conducted by the three VIN13 strains using Müller-Thurgau must with the *isomixed, closed* (**A**), *isomixed, open* (**B**), *static, closed* (**C**) and the *static, open* (**D**) fermentation styles. Fermentations were stopped once no further weight loss was recorded. As the ANKOM Rf system shown in A continuously measures the accumulated pressure within a fermentation, only datapoints corresponding to a 24-h period are shown in the graph. Error bars indicate the standard deviation of four samples.

**Figure 3:**
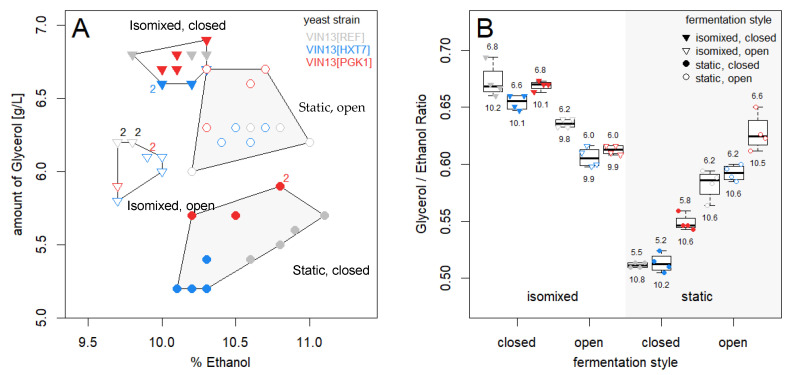
Relationship between the glycerol and ethanol levels in the wines produced under different fermentation styles. (**A**) Scatter plot with the percentage of ethanol plotted on the *x*-axis and the glycerol (in g/L) on the *y*-axis. Numbers indicate the presence and abundance of samples which showed similar amounts of both ethanol and glycerol, and hence, were plotted on top of one another. (**B**) is a box-and-whiskers plot showing the glycerol/ethanol ratio with the amount of glycerol [g/L] divided by ethanol content (%). For each VIN13 strain and fermentation condition, the average amount of glycerol [g/L] and ethanol content (%) are given above and below the box-and-whiskers plots, respectively. Colored points show the actual values obtained from each fermentation.

As outlined in the methods section, four different styles of fermentation were employed to ferment the same Müller-Thurgau must. As depicted in Figure 2, it took 7 days for all the isomixed fermentations to reach completion (sugar level below 5 g/L as determined by HPLC analyses) (Table S2). We used an ANKOM Rf system to measure the pressure built-up by CO_2_ production during fermentation; once no increases in pressure were observed, the fermentations were considered finished. It took 19 days for no weight-loss to be recorded for the static fermentations—either with or without an airlock. No significant differences in the vigor of the fermentation were observed between the VIN13 parental strain and the modified strains in the respective fermentation styles (Figure 3).

#### 3.2.2. Organic Acids

Apart from acetic acid, no noteworthy pattern between the three VIN13 strains within a specific fermentation style was observed in the tested organic acids (values shown in Table S2). The VIN13[PGK1p_ATF1] strain produced significantly less acetic acid compared to the other two strains, except in the static, closed fermentation style. The two main wine acids, namely tartaric acid and malic acid showed some degrees of fluctuation between the different styles. The tartaric acid concentration remained unchanged from the starting must in the static, open fermentations, whereas significant drops in the other styles were observed with the lowest levels measured in *the isomixed, closed* fermentation. The malic acid levels were markedly higher in the *isomixed, closed* fermentation compared to the other fermentation styles.

#### 3.2.3. Ethanol and Glycerol

**Figure 4:**
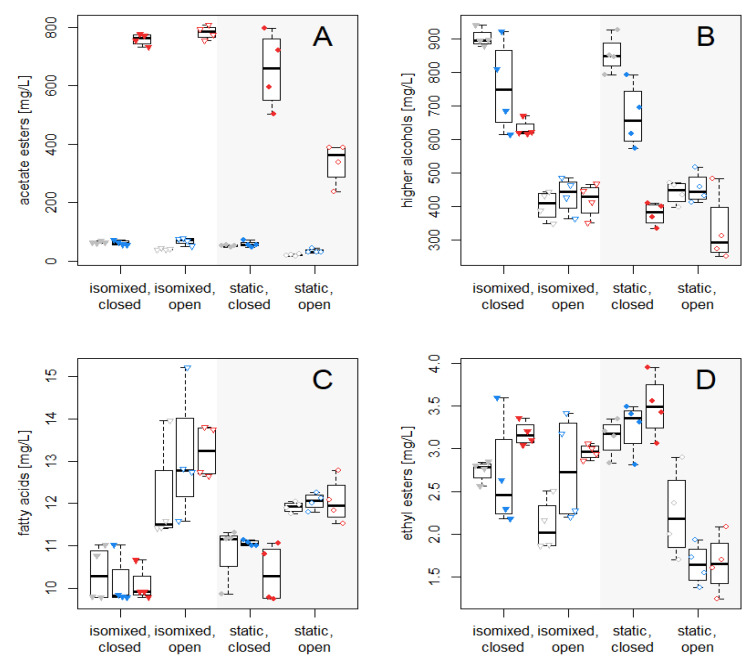
Effect of fermentation styles on the main groups of volatiles produced by the VIN13 strains. Box-and-Whiskers plots were drawn of the sum of the acetate esters (**A**), higher alcohols (**B**), fatty acids (**C**) and ethyl esters (**D**) measured in the final wines. Values of the individual components are shown in Table S3.

The two key alcohols in wine, namely glycerol and ethanol, were noticeably affected by the different fermentation styles, as shown in Table S2 and Figure 4. Both static-styled fermentations yielded similar ethanol levels of 10.5 ± 0.3% (static, closed) and 10.5 ± 0.08% (static, open). The isomixed fermentations produced lower ethanol levels compared to the static-styled fermentations, with the lowest being measured in the *isomixed, open* style (9.8 ± 0.06%). A decrease in ethanol did not necessarily correspond with a higher glycerol level and, as we show in Figure 4, the different fermentation styles grouped together with regards to their glycerol and ethanol levels. Interestingly, a marked increase in the glycerol levels was noted in the wines produced statically, but not in the isomixed, by the VIN13[PGK1p_ATF1] strain. This could be due to the prolonged exposure to drastically more acetate esters which are present in these fermentations compared to the VIN13[REF] and VIN13[HXT7p_ATF1] strains.

### 3.3. Volatile Compounds

We analyzed the acetate esters, higher alcohols, fatty acids and ethyl esters of the final wines with respect to the fermentation styles using GC-MS (Figure 4 and Table S3).

#### 3.3.1. Acetate Esters

**Figure 5:**
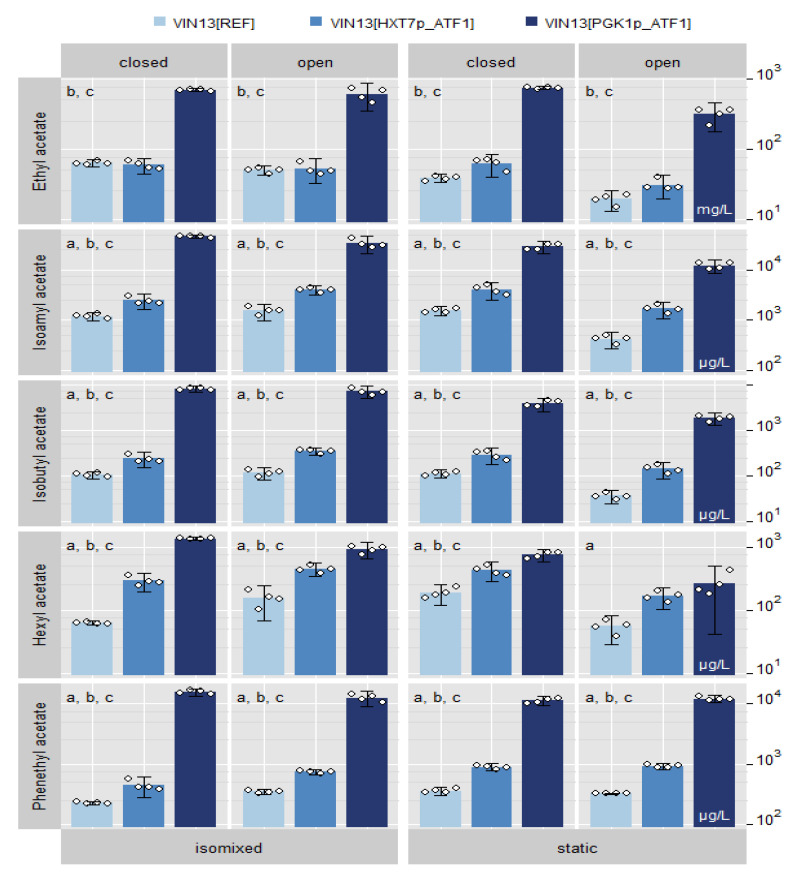
Effect of *HXT7* promoter on the expression of *ATF1* in a wine fermentation. Acetate ester levels of the final Müller-Thurgau wines fermented with the *ATF1*-overexpressing VIN13 strains produced from the isomixed and static fermentations. Values are in μg/L, except for ethyl acetate, which are in mg/L. The error bar represents 2 times standard deviation of a quadruplicate. Significance was assessed by linear contrasts with family-wise error rate of 0.05 for each acetate ester; letters a, b and c identify significant (*p* < 0.05) pairwise differences a: between VIN13[REF] and VIN13[HXT7p_ATF1], b: between VIN13[REF] and VIN13[PGK1p_ATF1], c: between VIN13[HXT7p_ATF1] and VIN13[HXT7p_ATF1].

We show the concentrations of the five common acetate esters in wine using the different fermentation styles in Figure 5. The concentrations are also shown in Table S3. As expected, the *PGK1p*-driven expression of *ATF1* resulted in a dramatic increase in all the different acetate ester levels, which is consistent with previous attempts [5,6]. Acetate ester levels obtained from *ATF1* expression modulation via *HXT7p* were markedly lower than in modulation via *PGK1p*. On average, *PGK1p*-driven *ATF1* expression yielded an approximately 20-fold increase of acetate ester levels compared to reference levels, with a maximum 76.7-fold increase (Isobutyl acetate in *isomixed, closed* fermentations). In contrast, on average, *HXT7p*-driven *ATF1* expression increased 1.5-fold with a maximum 3.7-fold increase (Hexyl acetate in *isomixed, closed* fermentation). The exception was ethyl acetate, where no significant difference was observed between wines made by VIN13[HXT7p_ATF1] and the reference strain VIN13[REF] under any of the four fermentation styles. With regard to the style of fermentation, of note was the markedly lower amounts of total acetate esters in the static, open style, compared to the other styles.

#### 3.3.2. Higher Alcohols

Only in the static, closed style of fermentation was there a significant drop in the total amount of higher alcohols due to the effect of the *ATF1* overexpression converting the alcohols to the respective acetate esters. In general, the closed fermentations yielded increased alcohol contents.

Levels compared to the open fermentations. Of note were the significantly higher phenylethanol levels—a highly desirable compound imparting a rose/flower aroma to the wine—in the samples prepared using the *isomixed, closed* method.

#### 3.3.3. Fatty Acids

The fatty acids measured were not affected by the modulation of the *ATF1*-expression within a specific fermentation style. There was, however, higher fatty acid content present within the open styles (both isomixed and static) which could be due to increased oxygen availability that could result in an increase in the synthesis of fatty acids.

#### 3.3.4. Ethyl Esters

No significant differences with regard to ethyl esters were observed in the different fermentation styles. Only in the *static, open* fermentation, a marginally lower trend in the overall levels of ethyl esters was observed. Interestingly, in all the fermentation styles, the ethyl propionate levels were markedly higher once the *ATF1* was strongly overexpressed by the *PGK1* promoter (Table S3 and Figure S1). Previous examinations of *ATF1* overexpression and *ATF1* characterization did not report on the ethyl propionate levels, and although the literature suggests that *ATF1* is not involved in ethyl ester synthesis, we propose that Atf1p might show affinity to propionyl-CoA (which is only one carbon longer than acetyl-CoA), although this claim remains to be confirmed [5,6,23].

### 3.4. Multivariate Analysis

Principal Component Analysis (PCA) of fermentations with unmodified *S. cerevisiae* VIN13 (VIN13[REF]) yielded 15 Principal-component axes (PCs) with nonzero eigenvalues (Figure S2). Variable loadings calculated with NIPALS from resampled data showed strong correlation to the variable loadings obtained via PCA of the correlation matrix: Pearson correlations > 0.91 for loadings on principal-component axes 1 to 3 when resampling was performed within fermentation styles, and > 0.96 when resampling was performed across the entire dataset (*n* = 16). We therefore considered the estimates obtained via PCA to be robust. According to the selection criteria for meaningful axes, ordination of samples on PCs 1, 2 and 3 was likely to represent major trends present in the data structure of the multivariate data table (see Tables S2 and S3). The cumulative proportion of total variation displayed on PCs 1 to 3 was 88.9% (76.7% on PCs 1 and 2). The ordination on higher axes was inspected visually and deemed not to be meaningful.

Multivariate homogeneity of variance was not rejected (F(3, 12) = 0.1295, *p* = 0.96). The stirring regimen accounted for approximately 55% of total variation (RDA, F(1, 12) = 49.109, *p* = 0.01, R^2^ = 0.55) and capping type for approximately 15% (RDA, F(1, 12) = 13.274, *p* = 0.01, R^2^ = 0.1498). There was a significant interaction between stirring regimen and capping type, accounting for approximately 16% of total variation (RDA, F(1, 12) = 14.219, *p* = 0.01, R^2^ = 0.16). The full model including all three terms accounted for 86% of total variation (RDA, F(3, 12) = 25.534, *p* = 0.01, R^2^ = 0.86).

**Figure 6:**
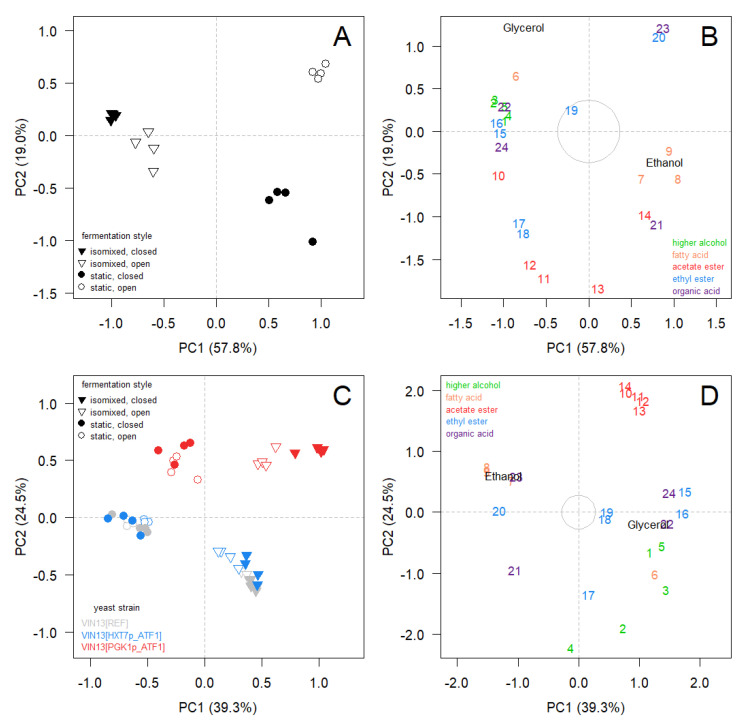
(**A**,**B**) Distance biplot of *Saccharomyces cerevisiae* VIN13[REF] fermentations in four different fermentation styles. (**C**,**D**) Distance biplot *Saccharomyces cerevisiae* strains VIN13[REF], VIN13[HXT7p_ATF1] and VIN13[PGK1p_ATF1] fermentations in four different styles. Sample ordination and analyte contribution have been separated for better readability. Distances among objects (fermentations) approximate Euclidean distance among them. Analytes (indicated by numbers in panels **B** and **D**, which contributed strongly to PC formation, are positioned at increasing distance to the equilibrium circle (grey at the centre). Analytes: higher alcohols isobutanol (1), isoamyl alcohol (2), 2-methyl butanol (3), hexanol (4), phenethyl alcohol (5); fatty acids: isovaleric acid (6), hexanoic acid (7), octanoic acid (8), decanoic acid (9); acetate esters ethyl acetate (10), isoamyl acetate (11), isobutyl acetate (12), hexyl acetate (13), phenethyl acetate (14); ethyl esters ethyl propionate (15), ethyl isobutyrate (16), ethyl butyrate (17), ethyl hexanoate (18), ethyl octanoate (19), ethyl decanoate (20); organic acids acetic acid (21), lactic acid (22), tartaric acid (23), malic acid (24); ethanol (25) and glycerol (26).

The interpretation of the proportion of variation explained by either stirring regimen or whether or not the vessel was sealed (capping type) is not straightforward, due to significant interactions between the two parameters. Observing an interaction was not surprising, given the fact that the experimental setup represented a four-level factor (fermentation style), rather than a pair of crossed two-level factors (stirring regimen X capping type). Nonetheless, sample ordination on distance biplot Figure 6A supports the hypothesis that stirring regimen (ordination on PC 1) had a much larger overall effect compared to capping type (i.e., compare proportions of variation shown on each PC). This was also true for fermentations with genetically modified strains after accounting for the statistical effects of *ATF1* expression type (approximately 35% by stirring regimen, and approximately 8% for capping type). The variable contributions shown in Figure 6B (cf. upper triangle in Figure S3) reveal that for the majority of analytes, levels were elevated in isomixed fermentations.

A principal component analysis (PCA) of data obtained from fermentations with all three *S. cerevisiae* VIN13 strains yielded 26 principal-component axes (PCs) with nonzero eigenvalues (Figure S4). According to both criteria for selecting meaningful axes, ordination of samples on PCs 1, 2 and 3 was likely to represent major trends present in the data structure of the multivariate data table (see Figures S4 and S5). The cumulative proportion of total variation of PCs 1 to 3 was 75.8%.

On PCs 1 and 2, ordination on higher axes (>5) was inspected visually and deemed not to be meaningful. *ATF1* expression modulation resulted in strong separation of fermentations with VIN13[PGK1p_ATF1] from fermentations with VIN13[REF] and VIN13[HXT7p_ATF1] on PC 2 due to increased amounts of acetate esters (Figure 5 and Figure 6C,D).

Similar to individual analyses of fermentations with VIN13[REF] (above), fermentations aligned along PC 1 according to stirring regimen. Multivariate homogeneity of variance was not rejected (F(11, 36) = 0.896, *p* = 0.55). Stirring regimen accounted for approximately 35% of total variation (RDA, F(1, 36) = 114.06, *p* = 0.01, R^2^ = 0.35); *ATF1* expression modulation accounted for approximately 26% (RDA, F(2, 36) = 41.873, *p* = 0.01, R^2^ = 0.258). This is was, however, largely due to *PGK1p*-driven *ATF1* expression. Capping type accounted for only approximately 8% (RDA, F(1, 36) = 26.474, *p* = 0.01, R^2^ = 0.08). Approximately 88.9% of total variation was accounted for by the full model (RDA, F(11, 36) = 26.26, *p* = 0.01, R^2^ = 0.889).

## 4. Discussion

The overexpression of *ATF1* with strong, constitutive promotors such as *PGK1*p or *TEF1*p in *S. cerevisiae* has previously been shown to dramatically increase the acetate ester content [7,8,27]. In our study, the fermentation of grape must with the VIN13[PGK1p_ATF1] strain resulted in an intense, and conceivably too strong, aroma. The mean average increase of desirable acetate esters, isoamyl acetate (banana, fruity), isobutyl acetate (fruity) and phenethyl acetate (floral, rose) in *isomixed, closed* fermentations was 61-fold. This was accompanied, however, by excessive production of ethyl acetate (10-fold increase). Ethyl acetate, despite having a comparatively high odor threshold of 7.5 mg/L [28], at high concentrations, imparts a strong chemical, nail polish aroma to a wine. Thus, increased levels of desirable acetate esters, while maintaining acceptable levels of ethyl acetate, would be preferable. We achieved this goal with VIN13[HXT7p_ATF1]. While maintaining levels of ethyl acetate that were similar to the control, isoamyl acetate, isobutyl acetate and phenethyl acetate increased moderately: 1.3- and 2.1-fold average increases in isomixed and static fermentations, respectively. It has been shown that a moderate increase (0.73-fold in acetate esters) of volatiles improves performance during sensory evaluation [29]. In addition, a reduction in acetic acid once *ATF1* is strongly overexpressed has been observed before, and could be explained by the fact that substantially more acetyl-CoA is directed toward the synthesis of acetate esters rather than being a precursor for acetate [7,27].

Promotor choice for expression is a critical parameter for expressing either homologous or recombinant genes. Whereas most GM wine yeast constructions involved the “usual suspects” of gene promoters involved in the glycolytic pathway, the performance of the promoter of *HXT7* has, to our knowledge, not been explored in grape must fermentation. This promoter is derepressible, is activated at low or depleted glucose concentrations, and has been the subject of many investigations for heterologous gene expression [30,31,32]. Although a thorough investigation in the expression profile of the *HXT7* promoter activity in a grape must fermentation is warranted, expression during the latter part of a primary fermentation could be useful for the production of compounds whose accumulation might have an inhibitory effect on the fermentation performance, as might be the case for terpenes.

Finding the correct fermentation conditions to make the ideal wine is a challenging enterprise for winemakers. We found that among the four fermentation styles we investigated, the effect of stirring regimen (static or isomixed fermentation) constituted the largest variation partitioning fraction: 55% and 35% of total variation could be explained by the stirring regimen when analyzing fermentations with VIN13[REF] and all fermentations, including the GM strains, respectively. Capping type (closed or open fermentation) accounted for only 16% and 8%, respectively.

Constant exposure to agitation is not common in practice, and few reports exist of experimental setups where constant stirring was applied to wine fermentations [33,34]. In the isomixed systems investigated in this study (i.e., an ANKOM Rf Gas production system and tall tube cylinders), the yeast is in constant suspension, unlike most static microvinifications with infrequent or no stirring, where most of the yeast cells eventually settle to the bottom of the fermentation vessel, as was the case in our static setups. We found that *isomixed, closed* (ANKOM Rf) and *isomixed, open* (tall-tube cylinders) fermentations were more alike, and that capping type had a much larger effect on static fermentations (ordination on PC 2, Figure 6A). This indicates that isomixing may be a means to increase the reproducibility of wine fermentations.

Most notably, and consistent with previous reports [34], isomixing provided an enormous decrease in fermentation duration in our study, i.e., 12 days less than a conventional fermentation. This was not accompanied with a general decrease of volatiles: for 8 out of 10 volatiles, the average difference observed between *static, closed* and *isomixed, closed* fermentations in our experiment was smaller than the average difference observed in fermentations of Chardonnay grape juice with 69 commercially available yeast strains [29], indicating that the volatile composition of isomixed wines is not altogether different from that of statically fermented wines.

Winemakers across the world are currently attempting to find ways to make wines with lower ethanol contents [35]. In our experiment, we observed reduced levels of ethanol and an increase of glycerol in the *isomixed, closed* fermentations. This contrasts with Rollero et al. [34], who observed no significant differences in ethanol levels when comparing static fermentations to fermentations with orbital agitation or with a stirring bar (130 rpm), albeit in an artificial must medium. In addition, further exploration, especially with mixed-cultured fermentations with non-*Saccharomyces* yeast with higher oxygen demands, might provide meaningful ways to address this question, as many mixed-culture fermentations with non-*Saccharomyces* mixed cultures have repeatedly been shown to lower the alcohol content in favor of glycerol production [36].

This report of isomixed wine fermentations warrants further investigation and larger scale trials to determine the fate of all of the wine components and include sensory panel analyses. Nevertheless, the sheer rapidity and even sensorial benefits showcase the utility of isomixing as a promising alternative to often time-consuming static fermentations. Short fermentations may not be useful for certain winemaking styles. On the other hand, the first fermentation for the production of a Grundwein to be used as a base for sparkling wine may benefit from isomixing.

## Supplementary Materials

The following are available online at https://www.mdpi.com/2304-8158/9/6/717/s1, Figure S1: Effect of *ATF1* expression modulation on ethyl propionate levels in a wine fermentation, Figure S2: Relative eigenvalues of 15 principal-component axes (PC 1 to PC 15), Figure S3: Distance Biplots of principal-component axes PC 1 to PC 3, Figure S4: Relative eigenvalues of 26 principal-component axes (PC 1 to PC 26), Figure S5: Distance biplots of principal-component axes PC 1 to PC 5, Table S1: Primers used in the study, Table S2: Final sugar, ethanol, glycerol and organic acids as determined for the three VIN13 strains in Müller-Thurgau wine from the different styles of fermentations, Table S3: Concentrations of the major volatiles of the final Müller-Thurgau wine product produced by VIN13-based strains from the isomixed and static fermentations as determined by GC-MS analysis.
