# Supplementary material for: Effect of Isomixing on Grape Must Fermentations of ATF1–Overexpressing Wine Yeast Strains
Source: Foods. 2020 Jun 2;9(6):717. doi: 10.3390/foods9060717 (PMC7353577; doi:10.3390/foods9060717)
Supplement: Supplementary file 1 [file foods-09-00717-s001.docx]

**Supplementary table S1:** **Primers used in the study.** The underlined sequence indicates site recognized by a restriction endonuclease

| Primer name (Restriction enzyme) | Sequence |
| --- | --- |
| *ScATF1*_F (*Pac*I)  *ScATF1*_R (*Asc*I)  *HXT7*prom_F (*Pac*I)  *HXT7*prom_R (*Pac*I) | CCCTTAATTAAATGAATGAAATCGATGAGAAAAATC  AAAGGCGCGCCCTAAGGGCCTAAAAGGAGAGCTTTG  CCCTTAATTAAAATAGTACTCTCATCGCTAAG  GGGGTTAATTAATTTTTGATTAAAATTAAAAAAAC |
| *PGK1*prom | TCATCAAGGAAGTAATTATC |

**Supplementary table S2.** **Final sugar, ethanol, glycerol and organic acids as determined for the three VIN13 strains in** **Müller-Thurgau wine from the different styles of fermentations*.*** All values are expressed in g/L and the “±” represents the standard deviation of a quadruplicate.

|  | residual sugars | Tartaric acid | Malic acid | Lactic acid | Acetic acid | Ethanol [%] | Ethanol | Glycerol |
| --- | --- | --- | --- | --- | --- | --- | --- | --- |
| **isomixed, closed** |  |  |  |  |  |  |  |  |
| VIN13[REF] | 0,0 ±0 | 3,3 ±0,1 | 2,0 ±0,1 | 0,3 ±0 | 0,3 ±0 | 10,1 ±0,2 | 101,3 ±2 | 6,8 ±0,1 |
| VIN13[PGK1p_ATF1] | 1,0 ±1 | 3,5 ±0,5 | 2,5 ±0,3 | 0,3 ±0 | 0,2 ±0 | 10,1 ±0,1 | 100,8 ±1 | 6,8 ±0,1 |
| VIN13[HXT7p_ATF1] | 0,5 ±2 | 3,3 ±0 | 2,2 ±0 | 0,3 ±0 | 0,3 ±0 | 10,1 ±0,2 | 101,0 ±2 | 6,6 ±0,1 |
| **isomixed, open** |  |  |  |  |  |  |  |  |
| VIN13[REF] | 0,0 ±0 | 2,8 ±0,1 | 1,6 ±0,1 | 0,2 ±0 | 0,3 ±0 | 9,8 ±0,1 | 97,4 ±0,7 | 6,2 ±0 |
| VIN13[PGK1p_ATF1] | 0,0 ±0 | 2,9 ±0,1 | 1,8 ±0,1 | 0,2 ±0,1 | 0,3 ±0 | 9,9 ±0,1 | 98,4 ±1 | 6,0 ±0,1 |
| VIN13[HXT7p_ATF1] | 0,0 ±0 | 2,9 ±0,1 | 1,8 ±0,1 | 0,2 ±0 | 0,3 ±0 | 9,9 ±0,1 | 98,5 ±1 | 6,0 ±0,1 |
| **static, closed** |  |  |  |  |  |  |  |  |
| VIN13[REF] | 1,4 ±0,5 | 3,5 ±0,1 | 1,5 ±0,1 | 0,1 ±0 | 0,4 ±0 | 10,8 ±0,2 | 108,3 ±2 | 5,5 ±0,1 |
| VIN13[PGK1p_ATF1] | 2,4 ±0,5 | 3,6 ±0,1 | 1,8 ±0,1 | 0,2 ±0 | 0,3 ±0,1 | 10,6 ±0,3 | 105,6 ±3 | 5,8 ±0,1 |
| VIN13[HXT7p_ATF1] | 4,9 ±0,3 | 3,5 ±0,2 | 1,6 ±0,1 | 0,0 ±0 | 0,4 ±0 | 10,2 ±0,1 | 102,0 ±1 | 5,2 ±0,1 |
| **static, open** |  |  |  |  |  |  |  |  |
| VIN13[REF] | 0,2 ±0,5 | 5,2 ±0,2 | 1,3 ±0 | 0,1 ±0 | 0,3 ±0 | 10,7 ±0,3 | 106,1 ±3 | 6,2 ±0,1 |
| VIN13[PGK1p_ATF1] | 0,4 ±0 | 5,2 ±0,1 | 1,7 ±0,1 | 0,2 ±0,1 | 0,2 ±0 | 10,5 ±0,2 | 104,5 ±2 | 6,6 ±0,2 |
| VIN13[HXT7p_ATF1] | 0,0 ±0,8 | 5,2 ±0,1 | 1,6 ±0,1 | 0,1 ±0 | 0,3 ±0 | 10,6 ±0,1 | 105,0 ±1 | 6,2 ±0,1 |

**Supplementary table S3.** **Concentrations of the major volatiles of the final Müller-Thurgau wine product produced by VIN13-based strains from the isomixed and static fermentations as determined by GC-MS analysis.** The compounds ethyl 2-hydroxy-4-methylvalerate and diethyl succinate were also determined but were either not detected or quantifiable in any sample. The “±” represents the standard deviation of a quadruplicate.

|  | Isomixed fermentation | | | | | | static fermentation | | | | | |
| --- | --- | --- | --- | --- | --- | --- | --- | --- | --- | --- | --- | --- |
|  | closed | | | open | | | closed | | | open | | |
|  | VIN13  [REF] | VIN13  [PGK1p_ATF1] | VIN13  [HXT7p_ATF1] | VIN13  [REF] | VIN13  [PGK1p_ATF1] | VIN13  [HXT7p_ATF1] | VIN13  [REF] | VIN13  [PGK1p_ATF1] | VIN13  [HXT7p_ATF1] | VIN13  [REF] | VIN13  [PGK1p_ATF1] | VIN13  [HXT7p_ATF1] |
| **Higher alcohols** |  |  |  |  |  |  |  |  |  |  |  |  |
| Isobutanol [mg/L] | 59 ±3 | 50 ±9 | 36 ±9 | 77 ±12 | 41 ±7 | 41 ±5 | 28 ±3 | 47 ±9 | 26 ±4 | 30 ±2 | 30 ±12 | 27 ±3 |
| Isoamyl alcohol [mg/L] | 547 ±15 | 320 ±16 | 422 ±84 | 530 ±30 | 154 ±20 | 386 ±51 | 259 ±32 | 241 ±27 | 286 ±35 | 285 ±22 | 144 ±51 | 269 ±35 |
| 2-Methyl butanol [mg/L] | 173 ±6 | 130 ±12 | 176 ±33 | 170 ±20 | 121 ±16 | 158 ±28 | 61 ±10 | 75 ±11 | 61 ±12 | 75 ±5 | 85 ±31 | 92 ±10 |
| Hexanol [µg/L] | 3078 ±135 | 424 ±22 | 2935 ±349 | 2240 ±174 | 285 ±30 | 1990 ±126 | 2086 ±75 | 363 ±36 | 1967 ±127 | 1916 ±86 | 269 ±24 | 1575 ±86 |
| Phenethyl alcohol [mg/L] | 122 ±7 | 133 ±4 | 122 ±20 | 75 ±5 | 62 ±9 | 84 ±14 | 55 ±7 | 56 ±12 | 61 ±4 | 51 ±5 | 72 ±17 | 66 ±4 |
| mean total [mg/L] | 903,6 ±28 | 632,9 ±25 | 758,9 ±136 | 854,2 ±56 | 378,0 ±34 | 670,0 ±96 | 403,8 ±43 | 419,4 ±51 | 434,5 ±54 | 441,7 ±33 | 330,5 ±105 | 455,1 ±46 |
| **Fatty acids** |  |  |  |  |  |  |  |  |  |  |  |  |
| Isovaleric acid [µg/L] | 1943 ±110 | 1909 ±104 | 1947 ±112 | 2212 ±195 | 1910 ±141 | 2110 ±65 | 1408 ±78 | 1438 ±30 | 1462 ±56 | 1690 ±48 | 1679 ±216 | 1814 ±119 |
| Hexanoic acid [mg/L] | 4,5 ±0,6 | 4,2 ±0,5 | 4,2 ±0,5 | 4,8 ±0,5 | 4,5 ±0,6 | 5,0 ±0,0 | 5,2 ±0,5 | 6,0 ±0,0 | 5,2 ±0,5 | 5,0 ±0,0 | 5,0 ±0,0 | 5,0 ±0,0 |
| Octanoic acid [mg/L] | 3,0 ±0,0 | 3,0 ±0,0 | 3,0 ±0,0 | 3,0 ±0,0 | 3,0 ±0,0 | 3,0 ±0,0 | 4,2 ±0,5 | 4,5 ±0,6 | 5,0 ±0,8 | 4,0 ±0,0 | 4,2 ±0,5 | 4,0 ±0,0 |
| Decanoic acid [µg/L] | 903 ±20 | 919 ±36 | 924 ±33 | 917 ±14 | 942 ±17 | 947 ±34 | 1192 ±247 | 1300 ±62 | 1377 ±223 | 1224 ±113 | 1127 ±62 | 1240 ±77 |
| mean total [mg/L] | 10,3 ±1 | 10,1 ±0 | 10,1 ±1 | 10,9 ±1 | 10,4 ±1 | 11,1 ±0 | 12,1 ±1 | 13,2 ±1 | 13,1 ±2 | 11,9 ±0 | 12,1 ±1 | 12,1 ±0 |
| **Acetate esters** |  |  |  |  |  |  |  |  |  |  |  |  |
| Ethyl acetate [EtAc] [mg/L] | 63 ±3 | 687 ±17 | 59 ±8 | 49 ±4 | 600 ±125 | 52 ±10 | 38 ±3 | 737 ±21 | 62 ±11 | 19 ±3 | 312 ±69 | 31 ±6 |
| Isoamyl acetate [µg/L] | 1191 ±119 | 46392 ±1821 | 2460 ±420 | 1547 ±283 | 33777 ±6508 | 3989 ±398 | 1543 ±152 | 28924 ±3788 | 4090 ±796 | 418 ±73 | 12194 ±1790 | 1684 ±307 |
| Isobutyl acetate [µg/L] | 107 ±9 | 8303 ±633 | 243 ±44 | 117 ±17 | 7227 ±1112 | 348 ±33 | 113 ±12 | 3947 ±645 | 294 ±59 | 37 ±6 | 1869 ±268 | 144 ±29 |
| Hexyl acetate [µg/L] | 62 ±2 | 1355 ±30 | 290 ±47 | 155 ±44 | 914 ±131 | 446 ±53 | 187 ±34 | 745 ±82 | 430 ±76 | 55 ±14 | 267 ±113 | 166 ±31 |
| Phenethyl acetate [µg/L] | 225 ±8 | 15573 ±1076 | 446 ±82 | 349 ±16 | 12492 ±1807 | 733 ±38 | 359 ±28 | 11230 ±930 | 885 ±60 | 324 ±4 | 12004 ±809 | 914 ±58 |
| mean total [mg/L] | 64,6 ±4 | 758,6 ±20 | 61,9 ±8 | 51,4 ±4 | 653,9 ±131 | 57,3 ±10 | 40,2 ±3 | 782,1 ±24 | 67,7 ±12 | 20,1 ±3 | 337,8 ±71 | 33,4 ±6 |
| [ex EtAc] mean total [mg/L] | 1,6 ±0 | 71,6 ±3 | 3,4 ±1 | 2,2 ±0 | 54,4 ±9 | 5,5 ±1 | 2,2 ±0 | 44,8 ±5 | 5,7 ±1 | 0,8 ±0 | 26,3 ±3 | 2,9 ±0 |
| **Ethyl esters** |  |  |  |  |  |  |  |  |  |  |  |  |
| Ethyl propionate [µg/L] | 609 ±46 | 1281 ±79 | 638 ±129 | 817 ±100 | 1028 ±87 | 535 ±47 | 135 ±15 | 312 ±19 | 112 ±23 | 89 ±16 | 238 ±64 | 115 ±24 |
| Ethyl isobutyrate [µg/L] | 26 ±2 | 46 ±2 | 26 ±2 | 30 ±4 | 49 ±6 | 30 ±2 | 0,0 ±0,0 | 0,0 ±0,0 | 0,0 ±0,0 | 0,0 ±0,0 | 0,0 ±0,0 | 0,0 ±0,0 |
| Ethyl butyrate [µg/L] | 262 ±18 | 141 ±24 | 232 ±30 | 192 ±10 | 116 ±15 | 210 ±22 | 216 ±12 | 173 ±11 | 275 ±50 | 90 ±12 | 60 ±9 | 103 ±12 |
| Ethyl hexanoate [µg/L] | 710 ±63 | 593 ±105 | 665 ±112 | 532 ±60 | 434 ±81 | 473 ±30 | 584 ±126 | 925 ±95 | 732 ±182 | 176 ±70 | 160 ±94 | 134 ±64 |
| Ethyl octanoate [µg/L] | 858 ±95 | 877 ±123 | 807 ±423 | 1216 ±192 | 1559 ±266 | 1532 ±195 | 784 ±105 | 1030 ±52 | 1049 ±294 | 1006 ±327 | 777 ±185 | 735 ±121 |
| Ethyl decanoate [µg/L] | 280 ±69 | 242 ±81 | 308 ±130 | 348 ±64 | 316 ±36 | 474 ±71 | 379 ±106 | 527 ±66 | 599 ±86 | 878 ±121 | 417 ±46 | 556 ±34 |
| mean total [mg/L] | 2,7 ±0 | 3,2 ±0 | 2,7 ±1 | 3,1 ±0 | 3,5 ±0 | 3,3 ±0 | 2,1 ±0 | 3,0 ±0 | 2,8 ±1 | 2,2 ±1 | 1,7 ±0 | 1,6 ±0 |


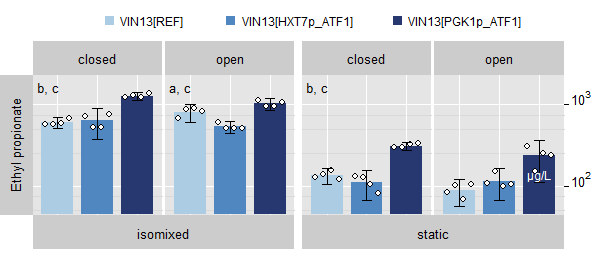


**Figure S1:** **Effect of *ATF1* expression modulation on ethyl propionate levels in a wine fermentation.** Values are in μg/L. The error bar represents ± 2 units of standard deviation of a quadruplicate. Significance was assessed by linear contrasts with family-wise error rate of 0.05; letters a, b and c identify significant (p < 0.05) pairwise differences a: between VIN13[REF] and VIN13[HXT7p_ATF1], b: between VIN13[REF] and VIN13[PGK1p_ATF1], c: between VIN13[HXT7p_ATF1] and VIN13[HXT7p_ATF1].


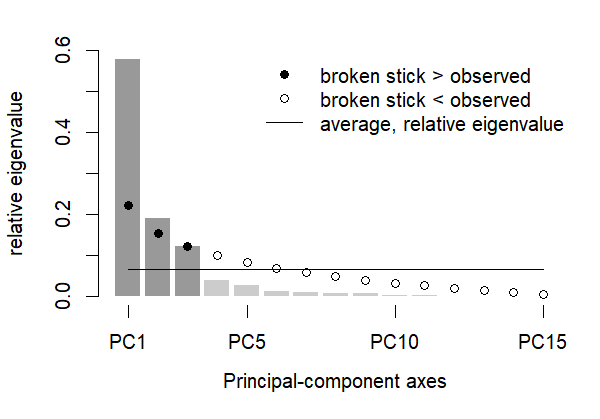


**Figure S2.** Relative eigenvalues of 15 principal-component axes (PC 1 to PC 15), obtained after performing Principal Component Analysis (PCA) on a data table holding observations on 26 analytes for 12 fermentations with *Saccharomyces* *cerevisiae* VIN13 in four different *fermentation styles* (quadruplicate fermentations in each style). Two selection criteria for identifying PC axes holding non-random ordination were applied: For PC axes 1 to 3. eigenvalues were larger than the mean of all 15 eigenvalues (Kaiser-Guttmann criterion) and, also, larger than expected by comparison to a broken stick model (bsm) of length 15 (cf. p. 448-449 in (Legendre & Legendre, 2012). Height of bars: relative eigenvalue for PC 1 to PC 15; dark grey: relative eigenvalues larger than Kaiser-Guttmann AND bsm criterion; horizontal line: mean eigenvalue (i.e. Kaiser-Guttmann threshold); points: expected eigenvalues obtained from a broken stick model of length 15; filled points: observed eigenvalues were larger than those obtained from the bsm; PC 1 to PC 3 pass both criteria; Kaiser-Guttmann criterion is known to be promiscuous; ordination on PC 4 and 5 was examined and did not show interesting patterns (e.g. fermentation style means were near zero; single fermentations showed they were outliers).


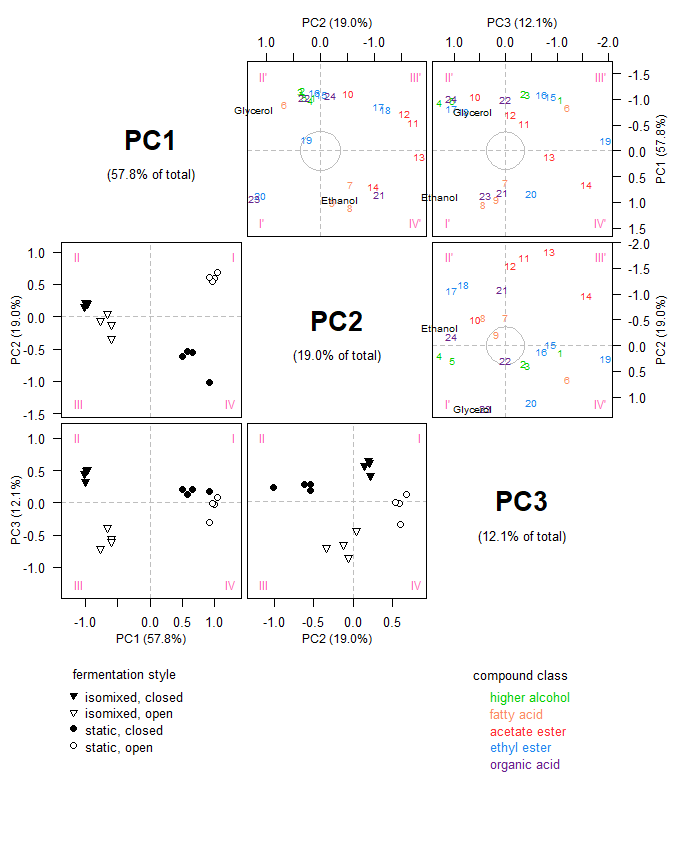


**Figure S3.** Distance Biplots of principal-component axes PC 1 to PC 3 (all 3 holding meaningful ordination according to selection criteria, see caption Figure S1), obtained after performing Principal Component Analysis (PCA) on a data table holding observations on 26 analytes for 12 fermentations with *Saccharomyces* *cerevisiae* VIN13 in four different *fermentation styles* (quadruplicate fermentations in each style). Plots showing descriptor contributions (upper-right array of panels) are mirrored: quadrant q’ corresponds to quadrant q of the corresponding plot in the bottom-left array of panels. Analytes: *higher alcohols* Isobutanol (1), Isoamyl alcohol (2), 2-Methyl butanol (3), Hexanol (4), Phenethyl alcohol (5); fatty acids: Isovaleric acid (6), Hexanoic acid (7), Octanoic acid (8), Decanoic acid (9); *acetate esters* Ethyl acetate (10), Isoamyl acetate (11), Isobutyl acetate (12), Hexyl acetate (13), Phenethyl acetate (14); *ethyl esters* Ethyl propionate (15), Ethyl isobutyrate (16), Ethyl butyrate (17), Ethyl hexanoate (18), Ethyl octanoate (19), Ethyl decanoate (20); *organic acids* Acetic acid (21), Lactic acid (22), Tartaric acid (23), Malic acid (24); Ethanol (25) and Glycerol (26)


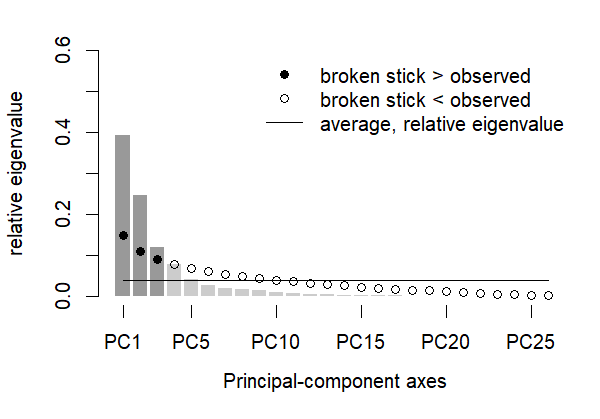


**Figure S4.** Relative eigenvalues of 26 principal-component axes (PC 1 to PC 26), obtained after performing Principal Component Analysis (PCA) on a data table holding observations on 26 analytes for 48 fermentations with different *Saccharomyces* *cerevisiae* VIN13 strains, including VIN13[REF], VIN13[HXT7p_ATF1] and VIN13[PGK1p_ATF1], in four different *fermentation styles* (quadruplicate fermentations in each style). PC 1 to PC 3 pass both, Kaiser-Guttmann criterion and broken stick model criterion. See caption Figure S1 for details.


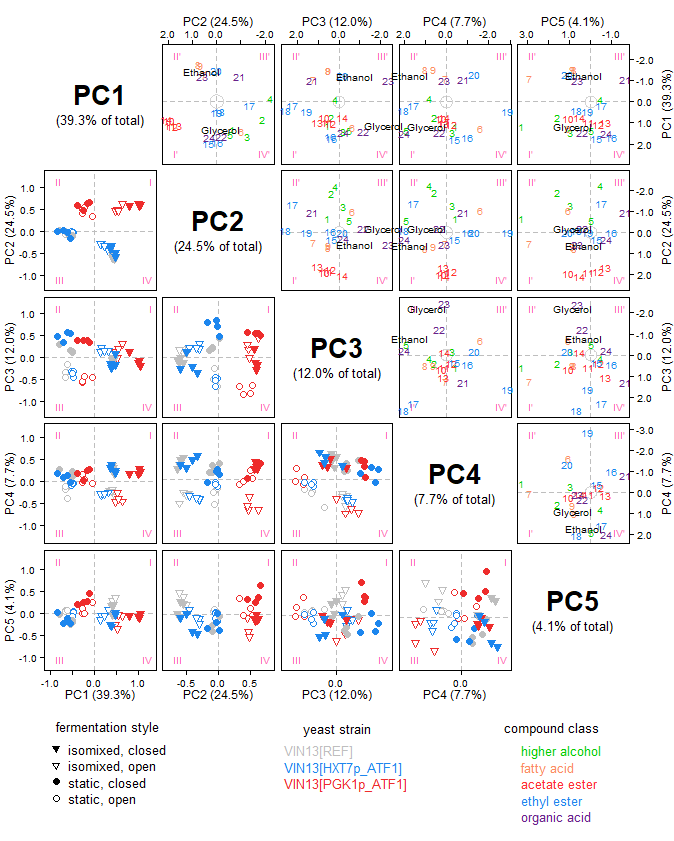


**Figure S5.** Distance biplots of principal-component axes PC 1 to PC 5 (all 5 holding meaningful ordination according to selection criteria, see caption Figure S1), obtained after performing Principal Component Analysis (PCA) on a data table holding observations on 26 analytes for 48 fermentations with different *Saccharomyces* *cerevisiae* VIN13 strains, including VIN13[REF], VIN13[HXT7p_ATF1] and VIN13[PGK1p_ATF1], in four different fermentation styles (quadruplicate fermentations in each style). Plots showing descriptor contributions (upper-right array of panels) are mirrored: quadrant q’ corresponds to quadrant q of the corresponding plot in the bottom-left array of panels. Analytes: *higher alcohols* Isobutanol (1), Isoamyl alcohol (2), 2-Methyl butanol (3), Hexanol (4), Phenethyl alcohol (5); fatty acids: Isovaleric acid (6), Hexanoic acid (7), Octanoic acid (8), Decanoic acid (9); *acetate esters* Ethyl acetate (10), Isoamyl acetate (11), Isobutyl acetate (12), Hexyl acetate (13), Phenethyl acetate (14); *ethyl esters* Ethyl propionate (15), Ethyl isobutyrate (16), Ethyl butyrate (17), Ethyl hexanoate (18), Ethyl octanoate (19), Ethyl decanoate (20); *organic acids* Acetic acid (21), Lactic acid (22), Tartaric acid (23), Malic acid (24); Ethanol (25) and Glycerol (26).
